# Supplementary material for: How Different Substitution Positions of F, Cl Atoms in Benzene Ring of 5-Methylpyrimidine Pyridine Derivatives Affect the Inhibition Ability of EGFRL858R/T790M/C797S Inhibitors: A Molecular Dynamics Simulation Study
Source: Molecules. 2020 Feb 18;25(4):895. doi: 10.3390/molecules25040895 (PMC7071101; doi:10.3390/molecules25040895)
Supplement: Supplementary file 1 [file molecules-25-00895-s001.pdf]

molecules

Supplementary Materials

How different substitution positions of F, Cl atoms in benzene ring of 5-methylpyrimidine pyridine derivatives affect the inhibition ability of EGFR$^{L858R/T790M/C797S}$ Inhibitors: A Molecular Dynamics Simulation Study

Jingwen E[1], Ye Liu[2], Shanshan Guan[3,4], Zhijian Luo[1], Fei Han[1], Weiwei Han[2], Song Wang[1*] and Hao Zhang[1*]

[1] Laboratory of Theoretical and Computational Chemistry, Institute of Theoretical Chemistry, Jilin University, Changchun 130023, People's Republic of China
[2] Key Laboratory for Molecular Enzymology and Engineering of Ministry of Education, School of Life Science, Jilin
[3] College of Food Engineering, Jilin Engineering Normal University, Changchun, Jilin 130052, China
[4] Key Laboratory of Molecular Nutrition at Universities of Jilin Province, Changchun, Jilin 130052, China
* Correspondence: ws@jlu.edu.cn (S. Wang); stringbell@jlu.edu.cn (H. Zhang); Tel.: +86-27-88498761

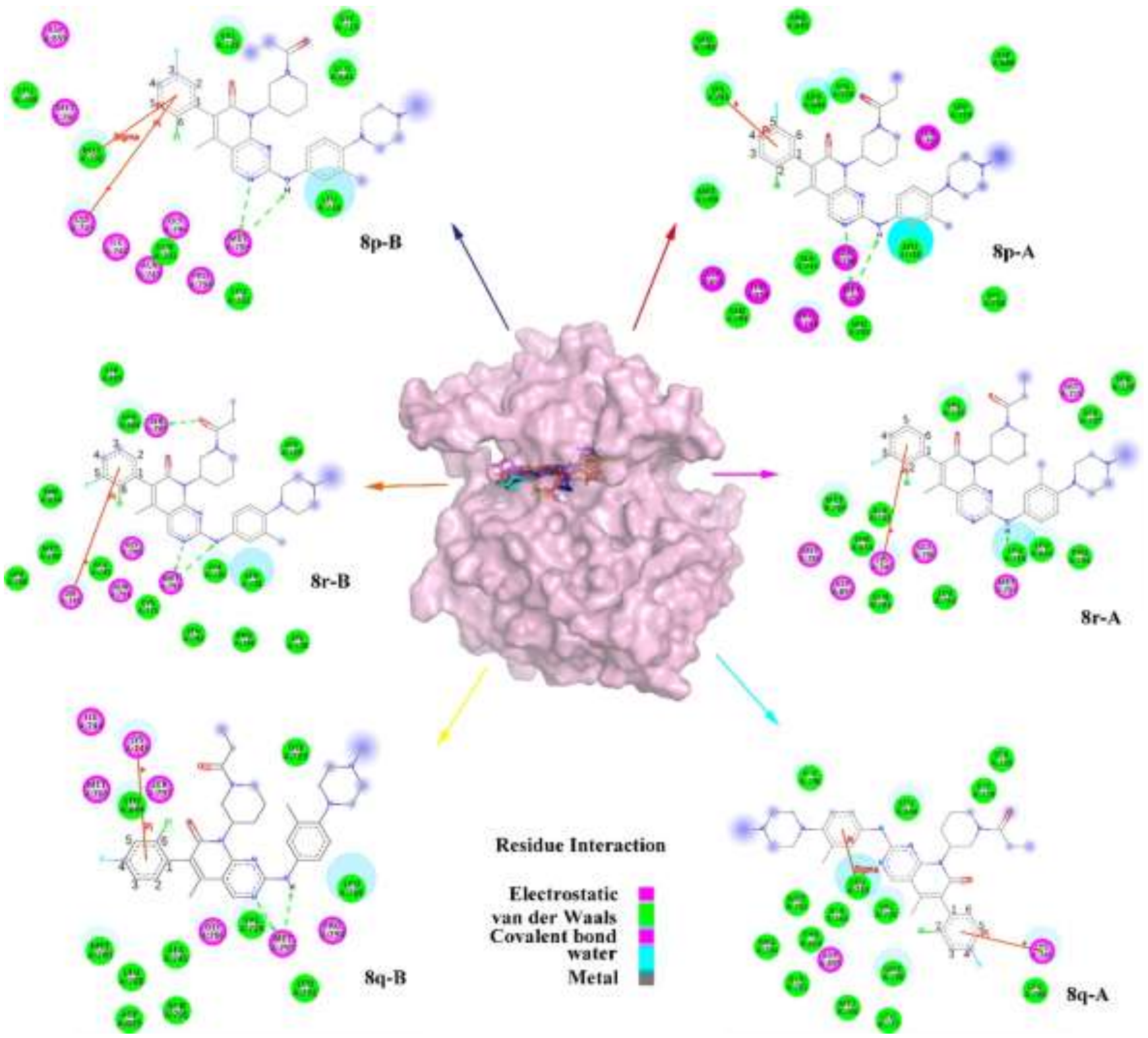

Figure S1. Optimal docking models of the six complexes: compound (**a**) 8r-B, (**b**) 8r-A (**c**) 8p-B (**d**) 8p-A (**e**) 8q-B and (**f**) 8q-A docked to EGFR™. Key residues and ligands are represented by stick models.

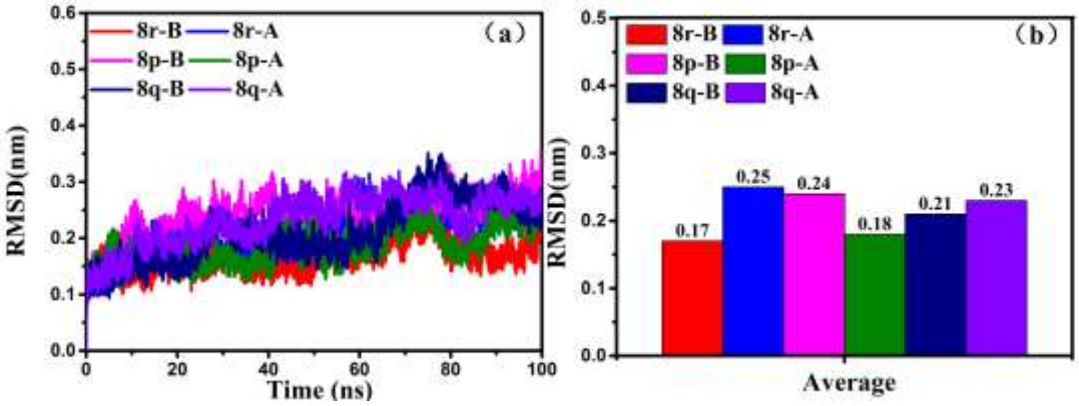

Figure S2. Stability analyses for EGFR™_8r-B(red), EGFR™_8r-A(blue), EGFR™_8p-B(magenta), EGFR™_8p-A(olive), EGFR™_8q-B(navy), EGFR™_8q-A(violet) complexes during the 100ns simulation. (**a**) RMSDs of the protein backbone, (**b**) Average RMSD values for the six systems.

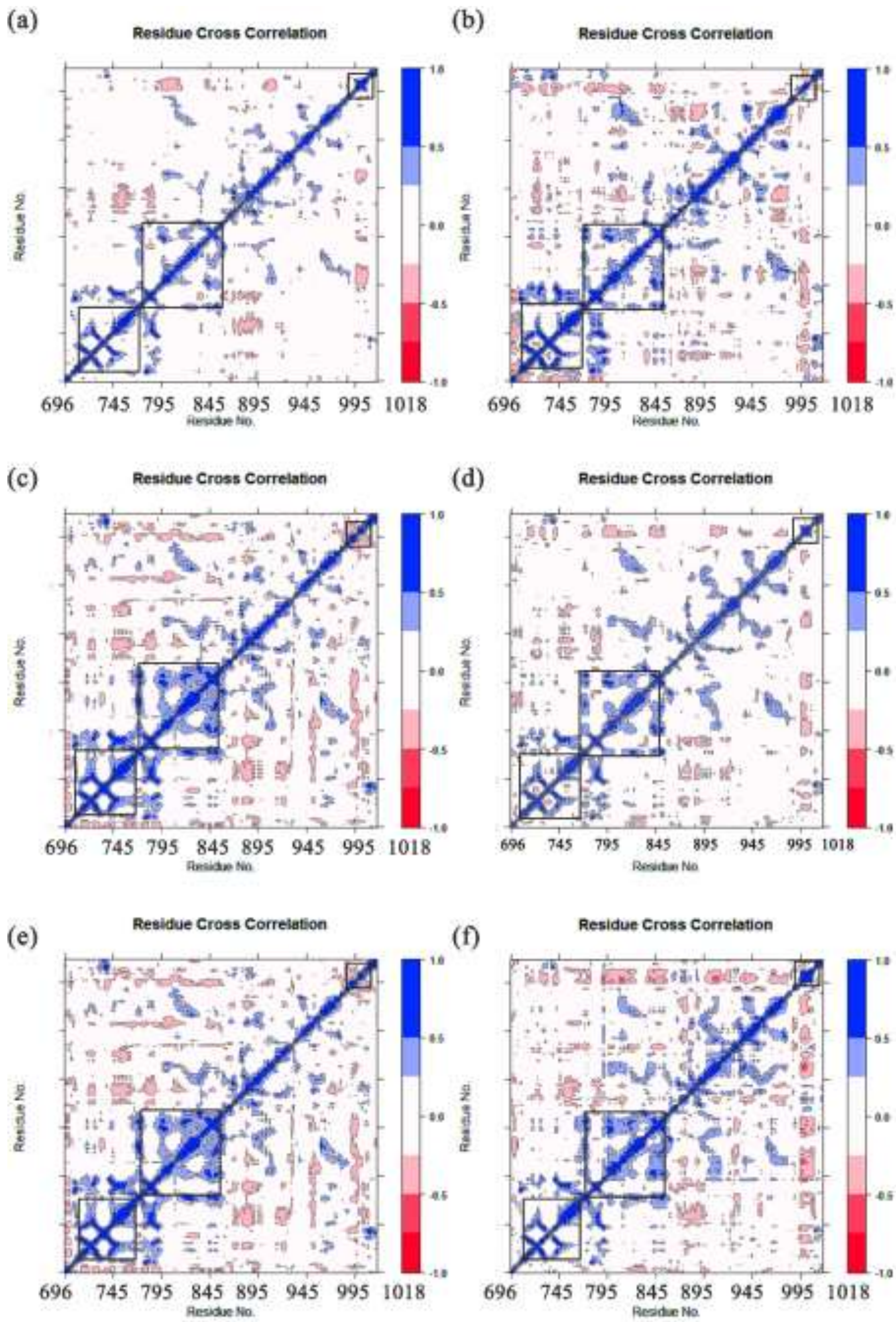

Figure S3. Cross-correlation matrix maps for the complex. (**a**) EGFR^{TM}-8r-B, (**b**) EGFR^{TM}-8r-A, (**c**) EGFR^{TM}-8p-B, (**d**) EGFR^{TM}-8p-A, (**e**) EGFR^{TM}-8q-B, and (**f**) EGFR^{TM}-8q-A.

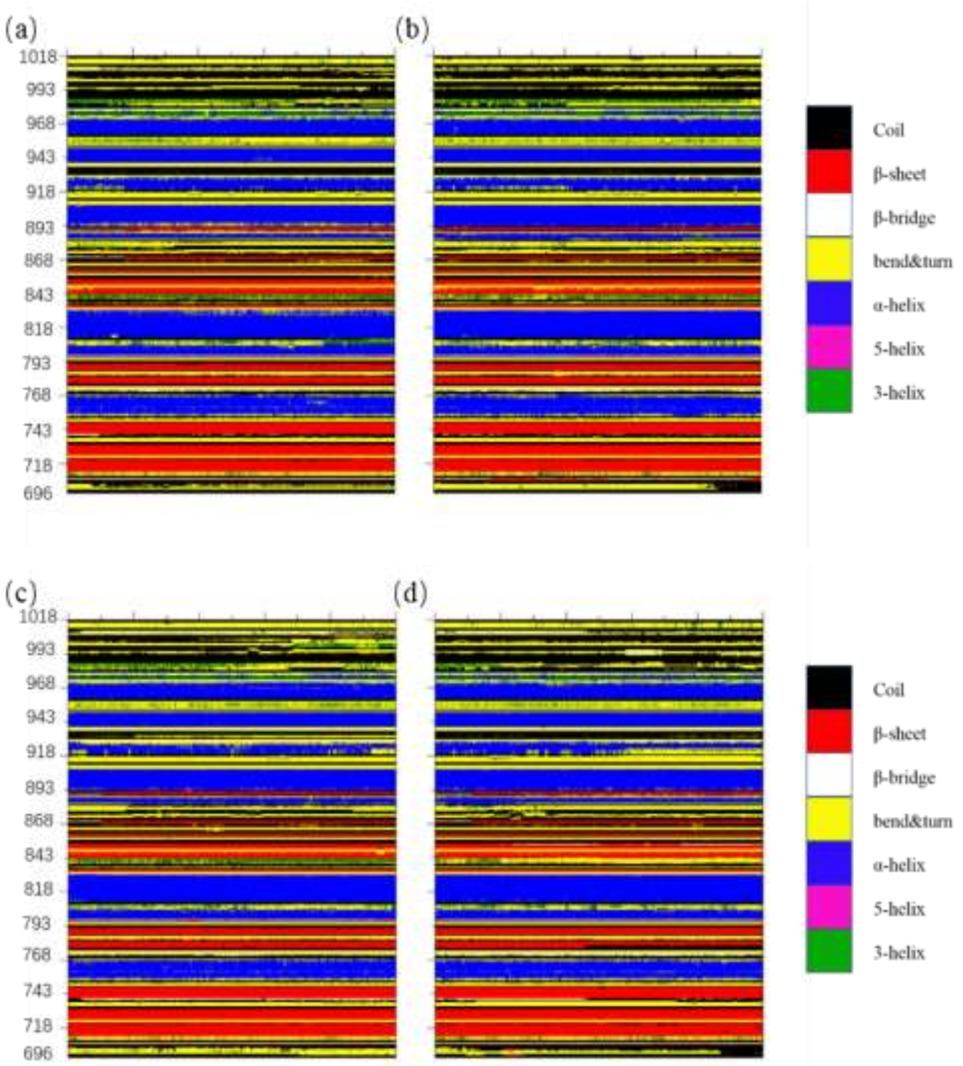

Figure S4. Comparison the differences of secondary structural differences between (a) 8p-B-bound, (b) 8p-A-bound, (c) 8q-B-bound, (d) 8q-A-bound proteins.

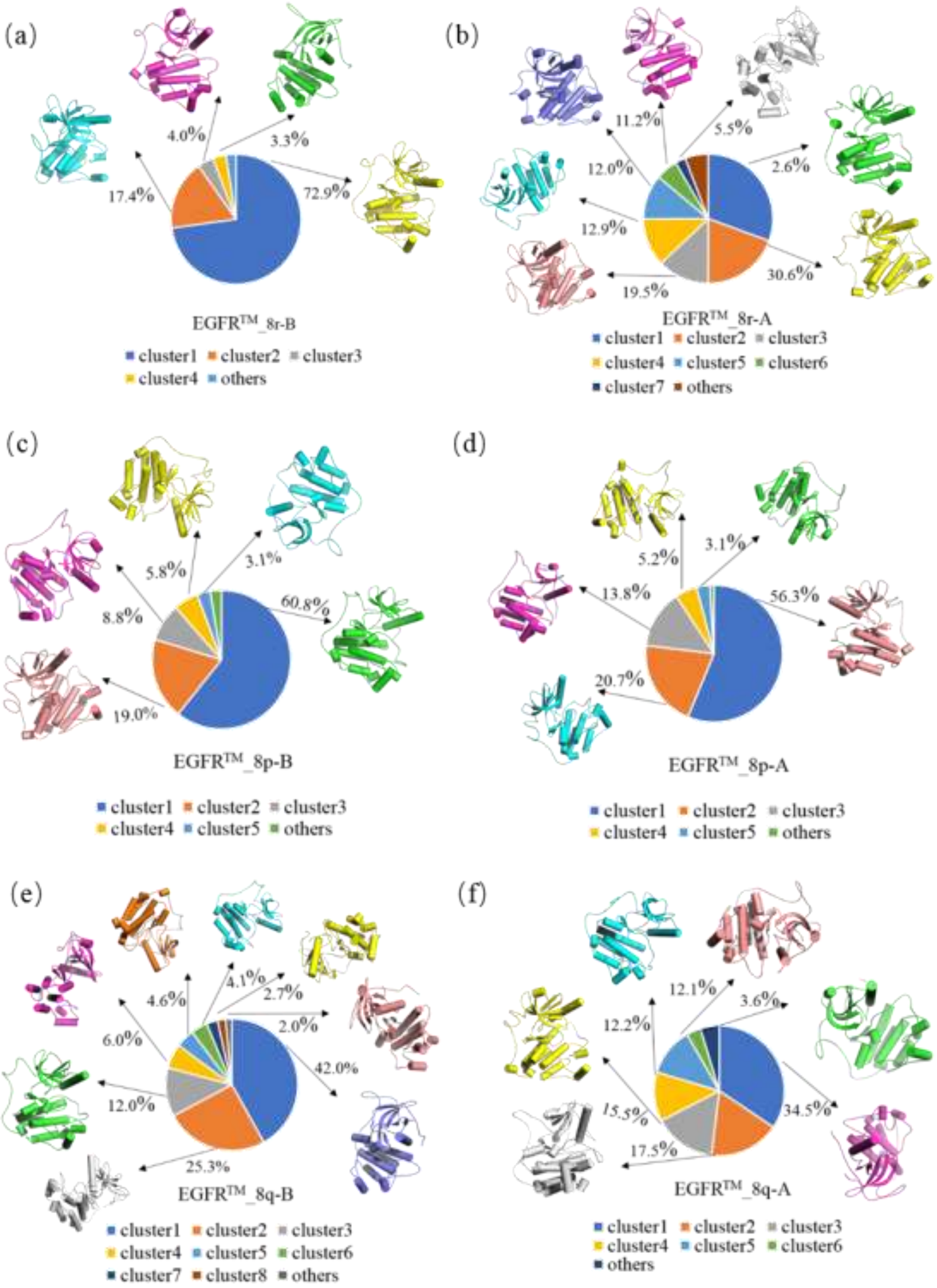

Figure S5. Structure based clustering analysis on equalized trajectory. Cluster centroids are shown and labeled. The clusters are shown others which percentage population were lower than 2%. (**a**) Complex EGFRTM_8r-B, (**b**) Complex EGFRTM_8r-A, (**c**) Complex EGFRTM_8p-B, (**d**) Complex EGFRTM_8p-A, (**e**) Complex EGFRTM_8q-B, (**f**) Complex EGFRTM_8q-A.

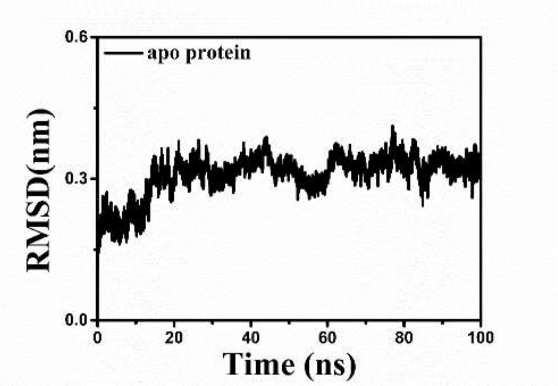

Figure S6. The optimized protein backbone atoms' RMSDs.

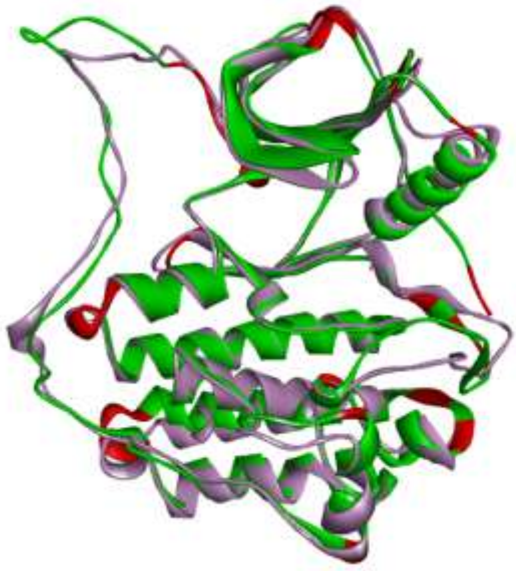

Figure S7. The comparison between the protein in 5EDP.pdb (green) and the optimized protein (purple). Different for two structure show as red region.

```
696    GEAPNQALLR ILKETEFKKI KVLGSGAFGT VYKGLWIPEG EKVKIPVAIK ELREATSPKA NKEILDEAYV MASVDNPHVC RLLGICLTST VQLIMQLMPF  795
5EDP              B  GGG EEE EEEEEE  SS EEEEEEEE   TTSBEEEEEE EE     HHH HHHHHHHHHH HHT   TTB   EEEEEEESSS EEEEEE   TT
Opt    TTT        B  TTS EEE EEEEEE  SS EEEEEEEE   TTSEEEEEEE EE     TTH HHHHHHHHHH HHS   TTB   EEEEE   SS  EEEEE   TT
796    GCLLDYVREH KDNIGSQYLL NWCVQIAKGM NYLEDRRLVH RDLAARNVLV KTPQHVKITD FGLAKLLGAE EKEYHAEGGK VPIKWMALES ILHRIYTHQS  895
5EDP    BHHHHHHHH GGG   HHHHH HHHHHHHHHH HHHHHTTEE  S    GGGEEE SSSS EEE      TT EE   SS   S EE   S      GGGS HHH HHH EE HHH
Opt     BHHHHHHHS TTT   HHHHH HHHHHHHHHH HHHHHTTEE  S    GGGEEE EETT EEE      TT EE   TT        B   S      TTTS THH HHT B  HHH
896    DVWSYGVTVW ELMTFGSKPY DGIPASEISS ILEKGERLPQ PPICTIDVYM IMVKCWMIDA DSRPKFRELI IEFSKMARDP QRYLVIQGDE RMHLPSPTDS  995
5EDP   HHHHHHHHHH HHHTTS   TT TT   HHHHHH HHHHT         TTB HHHHH HHHHHT SSG GGS   HHHHH HHHHHHHHSH HHHB   TTTT T
Opt    HHHHHHHHHH HHHTTS   TT TT   TTHHHH HHHTT         TTBTHHHHH HHHHHT SSG GGS   HHHHH HHHHHHTT G GGTB   TTTT T
996    NFYRALMDEE DMDDVVDADE YLI                                                                                      1018
5EDP              TTB  GGG
Opt               SSSS TTT
```

Figure S8. Comparison of secondary structure for constructed structure. H=α-helix, B=residue in isolated β-bridge, E=extended strand, participates in β-ladder, G=3-helix (3/10helix), I=5-helix (pi helix), T=hydrogen bonded turn, S=bend, Blank=loop or irregular.

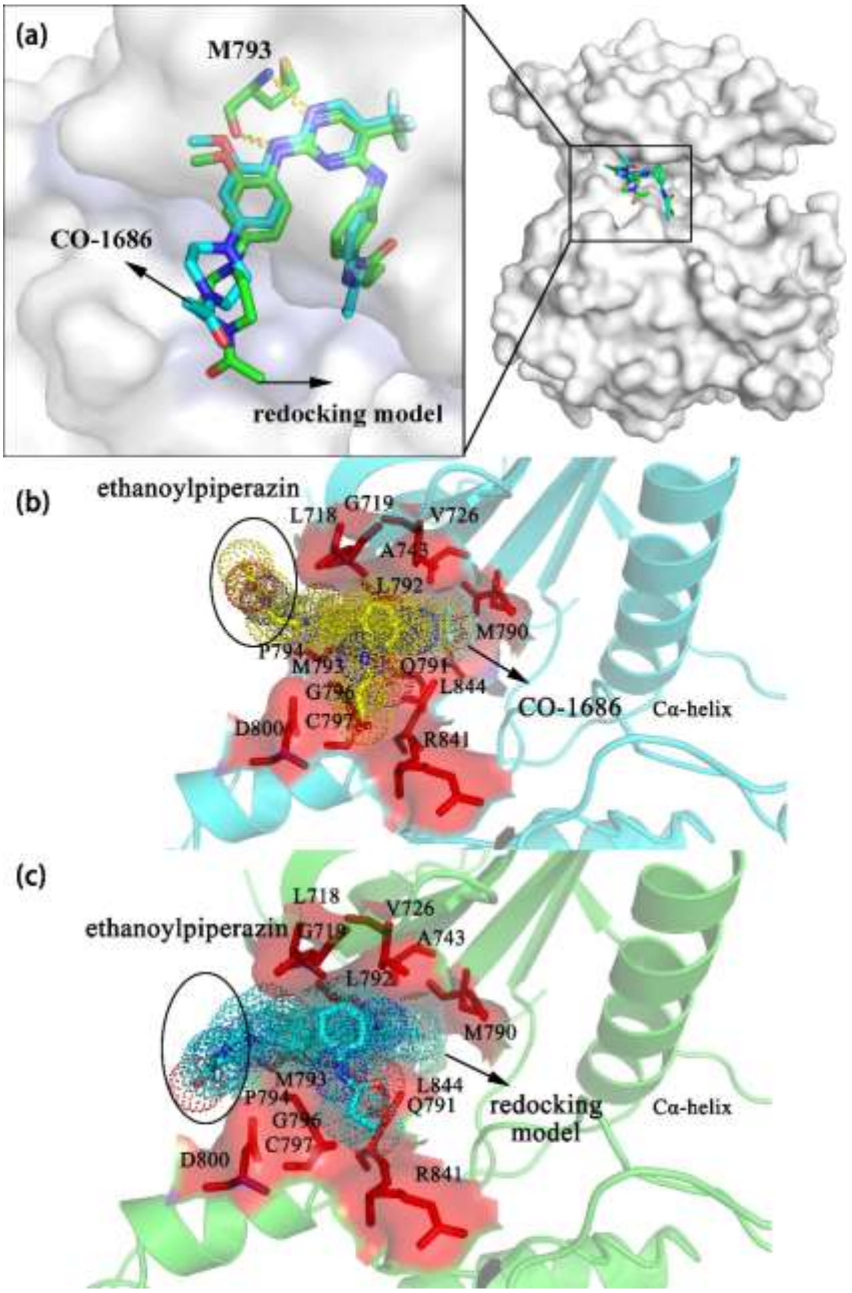

Figure S9. Redocking result for complex. (**a**) Overall comparison of crystal ligand CO-1686 (cyan stick) and redocking model (green stick). The main contributing residues (red stick and surface) of CO-1686-bound (**b**) and redocking model-bound (**c**) complexes during docking calculations.

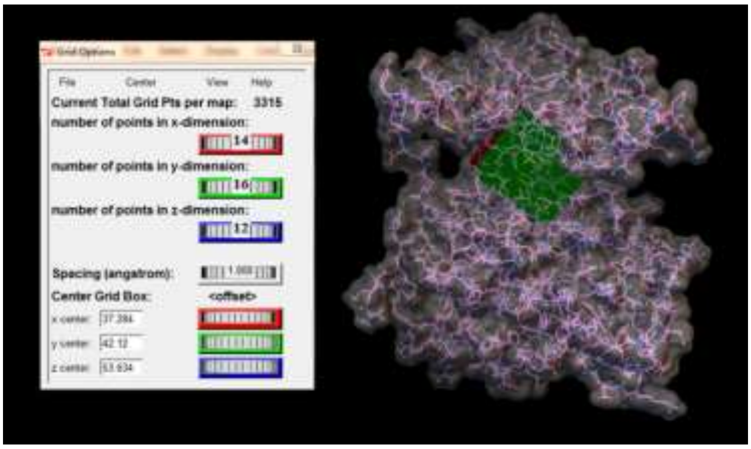

Figure S10. Docking grid for six inhibitors bound with EGFR™.
